# Predictors of problematic smartphone use among young adult lesbian, gay and bisexual individuals during the COVID-19 pandemic: a four-year follow-up study

**DOI:** 10.1186/s12888-023-05326-2

**Published:** 2023-12-05

**Authors:** Mei-Feng Huang, Yu-Ping Chang, Wen-Jiun Chou, Cheng-Fang Yen

**Affiliations:** 1 Department of Psychiatry, School of Medicine, College of Medicine, Kaohsiung Medical University Hospital, Kaohsiung Medical University, Kaohsiung, Taiwan; 2 School of Nursing, The State University of New York, University at Buffalo, New York, NY USA; 3 Department of Child and Adolescent Psychiatry, Chang Gung Memorial Hospital, Kaohsiung Medical Center, 32 Dapi Rd. Niaosong Dist, Kaohsiung, 83341 Taiwan; 4 School of Medicine, Chang Gung University, Taoyuan, Taiwan; 5 Department of Psychiatry, Kaohsiung Medical University Hospital, and School of Medicine College of Medicine, Kaohsiung Medical University, 100 Tzyou 1st Road, Kaohsiung, 80708 Taiwan; 6 College of Professional Studies, National Pingtung University of Science and Technology, Pingtung, Taiwan

**Keywords:** Smartphone, Gay, Lesbian, Bisexual, Sexual stigma, Psychological well-being

## Abstract

**Background:**

This 4-year follow-up study was conducted to evaluate the predictive effects of prepandemic individual and environmental factors on problematic smartphone use (PSU) among young adult lesbian, gay, and bisexual (LGB) individuals during the COVID-19 pandemic.

**Methods:**

Data on prepandemic PSU, demographics, sexual stigma (e.g., perceived sexual stigma from family members, internalized sexual stigma, and sexual microaggression), self-identity confusion (e.g., disturbed identity, unconsolidated identity, and lack of identity), anxiety, depression, and family support were collected from 1,000 LGB individuals between August 2018 and June 2019. The participants’ PSU was surveyed again after 4 years (between August 2022 and June 2023). The associations of prepandemic individual and environmental factors with PSU at follow-up were analyzed through linear regression.

**Results:**

In total, 673 (67.3%) participants completed the follow-up assessment. The severity of PSU significantly decreased after 4 years (p = .001). Before the incorporation of PSU at baseline into the analysis model, the results of the model revealed that high levels depressive symptoms (p < .001), disturbed identity (p < .001), and perceived sexual stigma from family members (p = .025) at baseline were significantly associated with PSU at follow-up. After the incorporation of PSU at baseline into the analysis model, the results of the model revealed that high levels PSU (p < .001) and depressive symptoms (p = .002) at baseline were significantly associated with PSU at follow-up.

**Conclusion:**

Interventions aimed at reducing the severity of PSU among LGB individuals should be designed considering the predictors identified in our study.

## Background

Smartphones have become an indispensable tool in modern life. People use smartphones to connect with others, get messages, have fun, learn, and go about their daily lives. However, smartphones have the ability to provide quick fun and close social interaction, which has led to an increasing dependence on smartphones. Individuals who have problematic smartphone use (PSU) experience compulsive smartphone use, tolerance to smartphone use, withdrawal symptoms if smartphones are unavailable, and functional impairment due to PSU [1]. A meta-analysis found that the global pooled prevalence estimate of PSU was 26.99% for PSU [2]. Another meta-analysis on 27 published studies demonstrated that PSU was associated with mental health problems (e.g., anxiety, sleep problems, and depression), physical problems (e.g., musculoskeletal problems), sedative lifestyles, and accidents [3]. Brand et al. proposed a Person-Affect-Cognition-Execution (I-PACE) model to illustrate the development and maintenance of problematic digital devices use [4]. The I-PACE model hypothesizes that problematic digital devices use is the consequence of interactions between predisposing factors (e.g., neurobiological and psychological constitutions), moderators (e.g., coping styles and Internet-related cognitive biases), and mediators (e.g., affective and cognitive responses to situational triggers in combination with reduced executive functioning); conditioning processes may strengthen these associations within an addiction process [4, 5]. Tan (2023) also proposed a stimulusorganismresponse–cognitiveadaptivenormative model to examine the drivers of habitual smartphone behavior and PSU [6].

Problematic digital devices use dramatically worsened during the coronavirus disease 2019 (COVID-19) pandemic [2]. People discontinue prepandemic socializing and recreational activities due to the lockdown or restriction of living areas in the COVID-19 pandemic; smartphones become the convenient tool for accessing information, entertainment and interaction with others. A systematic review and meta-analysis on 94 published studies revealed that the prevalence rate of PSU during the COVID-19 pandemic was 30.7%; in the lockdown periods, prevalence of problematic gaming and social media use were higher compared to non-lockdown periods [7]. Schoolchildren with a high level of problematic digital devices use had greater fear of COVID-19 [8] and psychological distress [9, 10] compared with those with a low level of problematic digital devices use, especial during COVID-19 school suspension [11]. Therefore, PSU is an emerging health issue during the COVID-19 pandemic that needs to be explored in depth.

Lesbian, gay, and bisexual (LGB) individuals are one of the populations at risk for PSU. LGB individuals experience public discrimination and prejudice due to their sexual orientation [12]. LGB individuals may conceal their sexual orientation and restrict their social interaction to avoid sexual stigma and bullying. Compared to the real world, the online world offers them a safer and more private place for obtaining entertainment and social interaction. Social media and dating applications on smartphone also provide the LGB individuals with a quick way to meet LGB friends and find sexual partners [13, 14]. However, a study in the United States found that problematic social media use among LGB young adults is associated with depression and low social support [15]. Two studies in Taiwan have also demonstrated that PSU among LGB individuals was associated with depressive and anxiety symptoms [16, 17]. A literature review evidenced that problematic Internet use is associated with health issues among the youth minority population [18]. The findings of previous studies highlight the significance of prevention and intervention of PSU in young adult LGB individuals.

LGB individuals were disproportionately affected by the COVID-19 pandemic in terms of psychological wellbeing compared with heterosexual individuals [19–21]. Disconnection with LGB communities can further worsen LGB individuals’ mental health. LGB individuals may rely more on smartphones to obtain social connection and entertainment and thus have a higher risk of PSU compared with heterosexual individuals. Furthermore, PSU can compromise LGB individuals’ ability to cope with the predicaments happened during the pandemic. Examining factors that can predict PSU in LGB individuals during the COVID-19 pandemic may provide a reference for developing intervention strategies to reduce the PSU risk.

According ecological system theory [22], there may be individual and environmental factors that increase the risk of PSU in LGB individuals. Regarding demographic characteristics, LGB individuals who are male and bisexual have greater PSU than those who are women and gay or lesbian, respectively [16]. Regarding environmental factors, sexual stigma was cross-sectionally associated with PSU severity in LGB individuals [16, 17]. However, no prospective study examined the prepandemic individual and environmental predictors of PSU in LGB individuals during the COVID-19 pandemic. In the Taiwanese Study of Sexual Stigma (T-SSS, in 2018 and 2019), the data of PSU, multiple types of sexual stigma (i.e., perceived sexual stigma from family members, sexual orientation microaggression, and internalized sexual stigma), self-identity confusion (i.e., disturbed identity, unconsolidated identity, and lack of identity), emotional problems (i.e., depressive and anxiety symptoms), and perceived family support in 1,000 LGB individuals living in Taiwan were collected [23–29]. Whether these prepandemic individual and environmental factors can predict the level of PSU in LGB individuals during the COVID-19 pandemic warrants study.

This 4-year follow-up study was conducted to investigate the predictive effects of prepandemic individual factors (e.g., demographics, sexual and gender identities, self-identity confusion, anxiety, depression, and PSU) and environmental factors (e.g., sexual stigma and perceived family support) on PSU during the COVID-19 pandemic in young adult LGB individuals. Based on the results of previous cross-sectional studies [16, 17]., we hypothesized that greater prepandemic sexual stigma was associated with greater PSU during the pandemic. Moreover, Internet provides young adults who have self-identity confusion with an environment to explore their personal values, beliefs, and goals [30, 31]; therefore, we hypothesized that greater prepandemic self-identity confusion was associated with greater PSU during the pandemic. A prospective study found the predictive effect of depression on PSU in Chinese adolescents [32]. A meta-analysis study also demonstrated a positive correlation between PSU and anxiety symptoms [33]. Therefore, we hypothesized that greater prepandemic depressive and anxiety symptoms were associated with greater PSU during the pandemic. Moreover, a prospective study demonstrated the predictive effect of low family support on problematic Internet use in Taiwanese adolescents [34]; therefore, we hypothesized that lower prepandemic family support was associated with greater PSU during the COVID-19 pandemic.

## Methods

### Participants and procedure

Both the criteria and methodology used in the T-SSS for recruiting participants at baseline have been described in previous studies [23–29]. In brief, a cohort of 1,000 individuals (500 men and 500 women) at baseline were recruited through online advertisements on social media platforms—including Facebook, Twitter, and LINE—and a bulletin board system from August 2018 to June 2019. The inclusion criteria were identifying as an LGB individual, being 20 to 30 years of age, and living in Taiwan. The exclusion criterion was having any form of impaired cognition that might interfere with the ability to complete a questionnaire.

Four years later (between August 2022 and June 2023), the same 1,000 individuals were contacted by text message and were invited to participate in a follow-up study. Those who responded to this message and agreed to participate received a blank consent form, a research questionnaire, and instructions for how to complete the questionnaire; they were also allowed to contact the research assistant for help if they had any problem understanding the questionnaire. Written informed consent was obtained from all participants. A total of three text messages were sent to the individuals to invite them to participate in the follow-up study, with a 1-month interval between each pair of messages. Those who responded to none of these messages were considered to have been lost to follow-up. This study was approved by the Institutional Review Board of Kaohsiung Medical University Hospital (KMUHIRB-F(I)-20210219).

### Measures

The outcome variable was PSU at follow-up. The predicting variables at baseline included prepandemic PSU, demographics, three types of sexual stigma, self-identity confusion, depression, anxiety, and perceived family support.

### Smartphone addiction inventory (SPAI)

The 26-item SPAI [1] was used to assess the participants’ self-reported severity of PSU in the three months prior to the baseline and follow-up assessments. The participants rated each item on a 4-point scale ranging from 1 (*totally disagree*) to 4 (*totally agree*), with a total score ranging from 26 to 104. A higher total score indicated a higher level of PSU. The Cronbach’s α coefficient of the SPAI in the present study was 0.96.

### Demographic characteristics

We collected data on participants’ sex, age, educational level (high school or lower vs. college or higher), sexual orientation (lesbian or gay vs. bisexual), and gender orientation (transgender or not).

### Homosexuality-related stigma scale (HRSS)

The 12-item HRSS was used to measure the levels of perceived sexual stigma from family members [35]. Each item was rated on a 4-point Likert scale with endpoints ranging from 1 (*strongly disagree*) to 4 (*strongly agree*). A higher total score indicated that the participant perceived a higher level of sexual stigma from family members [356]. The Cronbach’s α coefficient for this scale was 0.93 in this study.

### Measure of internalized sexual stigma for lesbians and gay men (TC-MISS-LG)

The 17-item traditional Chinese version [29] of the TC-MISS-LG [36] was used to assess each participant’s sexuality, identity, and level of social discomfort. Each item was rated on a 5-point Likert scale with endpoints ranging from 1 (*strongly disagree*) to 5 (*strongly agree*). A higher score indicated a greater level of internalized sexual stigma. The Cronbach’s α coefficient for this scale was 0.76 in this study.

### Sexual orientation microaggression inventory (SOMI)

The 19-item traditional Chinese version [25] of the SOMI [37] was used to assess microaggression in the dimensions of anti-gay attitudes and expressions, denial of homosexuality, and societal disapproval over 6 months among LGB individuals. Each item was rated on a 5-point Likert scale with endpoints ranging from 1 (*not at all*) to 5 (*almost every day*). A total higher score indicated a higher level of microaggression. The Cronbach’s α coefficient for this scale was 0.90 in the present study.

### Self-concept and identity measure (SCIM)

The traditional Chinese version [31, 38] of the 27-item SCIM was used to assess the level of current self-identity confusion [39, 40]. The SCIM assesses three dimensions of self-identity confusion, including disturbed identity, unconsolidated identity, and lack of identity. Items are rated on a 7-point rating scale ranging from 1 (*strongly disagree*) to 7 (*strongly agree*). A higher total score indicates a higher tendency for self-identity confusion. Cronbach’s alpha of the SCIM was 0.79 in the present study.

### Center for epidemiologic studies depression scale (CES-D)

The 20-item Mandarin Chinese version [41] of the CES-D [42] was used to assess the frequency of depressive symptoms within the preceding month. Each item was rated on a 4-point Likert scale with endpoints ranging from 1 (*rarely or none of the time*) to 4 (*most or all of the time*). A higher total score indicated more severe depression. The Cronbach’s α coefficient for this scale was 0.91 in this study.

### State-trait anxiety inventory (STAI)

The Mandarin Chinese version [43] of the STAI [44] consists of 20 items embedded in a single factor of anxiety. All the STAI items are assessed on a 4-point Likert scale, where a score of 1 indicates almost never and a score of 4 almost always. A higher STAI score indicates higher levels of anxiety [44]. The Cronbach’s α coefficient for this scale was 0.87 in this study.

### Adaptability, partnership, growth, affection, and resolve (APGAR) index

The Chinese version [45] of the APGAR Index [46] was used to assess the participants’ perceived support from their families. Each item was rated on a 4-point Likert-type scale with endpoints ranging from 1 (*never*) to 4 (*always*). The total scores for the Family APGAR Index range from 5 to 20, with higher total scores indicating higher levels of perceived support from family. In our study, the Cronbach’s α values for the Family APGAR Index were 0.94.

### Data analysis

All statistical analyses were conducted using SPSS (version 24.0; SPSS, Chicago, IL, USA). We employed descriptive statistics to summarize and analyze the participants’ data. The distributions of continuous variables were further tested for skewness and kurtosis to assess their level of departure from a normal distribution, and the results (i.e., absolute values of < 3 for skewness and < 10 for kurtosis) did not reveal any severe deviation [47].

This study detected the associations of prepandemic demographic characteristics, sexual stigma, self-identity confusion, depression, anxiety, perceived family support, and PSU with PSU at follow-up in two stages. In the first stage, the bivariate linear regression analysis involved the entry of only one independent variable at baseline for each time to detect their associations with PSU at follow-up. In the second stage, factors that were significantly associated with PSU in the bivariate linear regression analysis models were further included into a multivariate linear regression model to identify their associations with PSU at follow-up. Regarding the level of collinearity, the values of variance inflation factor ranged between 1.241 and 3.286; the values of tolerance ranged between 0.304 and 0.806; all values of eigenvalue were larger than 0.01; and the value of condition index was 29.862. The results indicated no problem of collinearity [48]. In order to understand what prepandemic factors were predictive of PSU severity at follow-up in the absence of the influence of prepandemic PSU, we used two regression analyses (one including and another not including prepandemic PSU as an independent variable) to identify the predictors of PSU at follow-up. Stepwise linear regression was performed to select most significant factors in the model. A *p* value of < 0.05 was regarded as significant.

## Results

In total, 673 (67.3%) participants responded to the invitation and agreed participating the follow-up study, 167 (16.7%) responded to the invitation but refused participating in the follow-up study, and 160 (16.0%) did not respond to the invitation. No differences in gender (χ^2^ = 0.005, *p* = .946), age (*t* = 1.890, *p* = .059), sexual orientation (χ^2^ = 2.087, *p* = .149), and gender orientation (χ^2^ = 15.767, *p* < .001) were found between those received and did not receive the follow-up survey; however, those who did not receive the follow-up survey were more likely to have an education level of high school or below (χ^2^ = 15.767, *p* < .001).

Table 1 shows participants’ demographics, sexual stigma, self-identity confusion, depressive and anxiety symptoms, perceived family function, and PSU. The gender distribution was relative similar (50.1% were men and 49.9% were women). The participants had a mean age of 24.8 years (standard deviation [SD] = 2.9 years) at baseline, and most of them had a college degree or higher (*n* = 618; 91.8%). More than half of the participants (*n* = 373; 55.4%) were gay or lesbian, and 19 participants (2.8%) identified themselves as transgender. The mean perceived sexual stigma from family members on the HRSS was 26.8 (SD = 6.3); the mean internalized sexual stigma on the MISS was 35.6 (SD = 11.5); and the mean sexual orientation microaggression on the SOMI was 42.3 (SD = 11.3). The mean level of disturbed identity on the SCIM was 37.5 (SD = 9.7), unconsolidated identity was 29.4 (SD = 8.7), and lack of identity was 19.2 (SD = 7.5). The mean score of depressive symptoms on the CES-D was 18.9 (SD = 11.3). The mean score of anxiety symptoms on the STAI was 41.2 (SD = 12.7). The mean perceived family function on the APGAR Index was 13.6 (SD = 3.6). The mean PSU on the SPAI was 61.9 (SD = 14.6) at baseline and 58.9 (SD = 17.4) at follow-up. The severity of PSU significantly decreased four years later (paired *t* = 5.351, *p* < .001).

**Table 1:** Demographics, Sexual Stigma, Self-Identity Confusion, Anxiety, Depression, Family Support, and PSU of Participants (N = 673)

Variable	n (%)	Mean (SD)	Range
Gender			
Women	336 (49.9)		
Men	337 (50.1)		
Age at baseline (year)		24.8 (2.9)	20–30
Education level			
High school or below	55 (8.2)		
College or above	618 (91.8)		
Sexual orientation			
Bisexual	300 (44.6)		
Gay or lesbian	373 (55.4)		
Transgender	19 (2.8)		
Perceived familial sexual stigma on the HRSS		26.8 (6.3)	10–40
Internalized sexual stigma on the MISS		35.6 (11.5)	17–76
Microaggression on the SOMI		42.3 (11.3)	19–78
Disturbed identity on the SCIM		37.5 (9.7)	13–67
Unconsolidated identity on the SCIM		29.4 (8.7)	10–64
Lack of identity on the SCIM		19.2 (7.5)	6–42
Anxiety symptoms on the STAI		41.2 (12.7)	20–79
Depressive symptoms on the CES-D		18.9 (11.3)	0–57
Family support on the APGAR Index		13.6 (3.6)	5–20
PSU on SPAI			
At baseline		61.9 (14.6)	26–101
At follow-up		58.9 (17.4)	26–101

APGAR Index: Adaptability, Partnership, Growth, Affection, and Resolve Index; CES-D: Center for Epidemiologic Studies Depression Scale; HRSS: Homosexuality-Related Stigma Scale; MISS: Measure of Internalized Sexual Stigma for Lesbians and Gay Men; SOMI: Sexual Orientation Microaggression Inventory; STAI: State-Trait Anxiety Inventory; SCIM: Self-Concept and Identity Measure; SPAI: Smartphone Addiction Inventory

Table 2 presents the results of bivariate linear regression analysis examining the individual associations of demographic characteristics, sexual stigma, self-identity confusion, depressive and anxiety symptoms, perceived family function, and PSU at baseline with PSU at follow-up. Three types of sexual stigma, three types of self-identity confusion, depressive and anxiety symptoms, and PSU at baseline were significantly associated PSU at follow-up. Lower perceived family function at baseline was significantly associated PSU at follow-up.

**Table 2:** Associations of Factors with PSU at Follow-Up: Bivariate Linear Regression Analysis

Variable	B (SE)	*p*
Gender	1.905 (1.340)	0.155
Age	0.347 (0.229)	0.131
Education level^a^	–1.898 (2.448)	0.438
Sexual orientation^b^	–1.008 (1.349)	0.455
Transgender	4.061 (4.047)	0.316
Perceived familial sexual stigma	0.414 (0.106)	< 0.001
Internalized sexual stigma	0.274 (0.057)	< 0.001
Sexual orientation microaggression	0.266 (0.058)	< 0.001
Disturbed identity	0.419 (0.067)	< 0.001
Unconsolidated identity	0.396 (0.076)	< 0.001
Lack of identity	0.635 (0.086)	< 0.001
Anxiety symptoms	0.321 (0.051)	< 0.001
Depressive symptoms	0.421 (0.057)	< 0.001
Family support	–0.475 (0.185)	0.012
PSU at baseline	0.699 (0.035)	< 0.001

^a^: high school or below as the reference; ^b^: bisexual as the reference

Factors that were significantly associated with PSU were further included in a stepwise linear regression model (Table 3). Before the incorporation of PSU at baseline into the analysis model, the results of the model revealed that high levels depressive symptoms (*p* < .001), disturbed identity (*p* < .001), and perceived sexual stigma from family members (*p* = .025) at baseline were significantly associated with PSU at follow-up. After the incorporation of PSU at baseline into the analysis model, the results of the model revealed that high levels PSU (*p* < .001) and depressive symptoms (*p* = .002) at baseline were significantly associated with PSU at follow-up.

**Table 3:** Predictors of PSU at Follow-up: Stepwise Multivariate Linear Regression Analysis

Variable	Model I		Model II	
	B (SE)	*p*	B (SE)	*p*
Depressive symptoms	0.310 (0.062)	< 0.001	0.149 (0.049)	0.002
Disturbed identity	0.254 (0.072)	< 0.001	–	–
Familial sexual stigma	0.235 (0.104)	0.025	–	–
PSU	–	–	0.697 (0.038)	< 0.001
F	25.111		209.566	
*p*	< 0.001		< 0.001	
Adjusted R^2^	0.097		0.383	

## Discussion

The present study found that the severity of PSU at follow-up significantly decreased compared with the prepandemic severity among LGB individuals. This finding is inconsistent with the findings of other studies examining the changes of digital devices use [2, 49]. For example, a 6-month follow-up study that the severity of problematic Internet use among children and adolescents significantly increased during the COVID-19 pandemic [49]. The present study examined the changes of the severity of PSU among young adults during a 4-year period; the participants may have significant growth in mental maturity and increase the ability to self-control smartphone use. However, there was a proportion of LGB individuals having a high severity of PSU measured by SPAI. For example, the total score of the SPAI ranged from 26 to 104, and 15.3% of the participants reported a total score of 78 (three-quarters of the total score) or higher on the SPAI. Moreover, the severity of PSU before the pandemic significantly predicted that of PSU after 4 years, indicating that LGB individuals with high prepandemic PSU tend to maintain their pattern of smartphone use during the COVID-19 pandemic. The result shows the need for early prevention of PSU among LGB individuals.

Our findings demonstrated that high levels depressive symptoms, disturbed identity, and perceived sexual stigma from family members at baseline predicted PSU at follow-up. LGB individuals have a higher risk of depressive disorders than do heterosexual individuals [50]. Both sexual minority stress [51] and intraminority LGB community stress [52, 53] result in high depressive symptoms in LGB individuals. LGB individuals with depressive symptoms may overuse smartphones to obtain entertainment and online social support for relieving negative emotion. Depression may also compromise LGB individuals’ ability to control their smartphone use. Therefore, depressive symptoms can predict the severity of PSU. Because PSU can worsen the individuals’ sleep rhythm, physical health, and the ability to cope with stress in the real world, PSU and depression may form a vicious cycle. This study highlights the necessity of early detection and intervention of depressive symptoms for preventing PSU.

This study demonstrated that prepandemic high disturbed identity predicted PSU four years later among LGB individuals. The individuals with a maturated self-identity have clear goals in life, is willing to make commitments, and make efforts to complete developmental tasks [54], whereas the individuals with disturbed identity tend to acquire the thoughts, feelings, and beliefs of others in adulthood [55]. Smartphones provide the individuals with disturbed identity the opportunities to assess the thoughts, feelings, and beliefs of others in the online world; therefore, the individuals with disturbed identity have a high demand for smartphones use and develop PSU gradually.

Our findings revealed that high prepandemic perceived sexual stigma from family members predicted PSU at follow-up among LGB individuals. Family is the basic and primitive microsystem in which the individuals are nourished [22]. LGB individuals who perceive sexual stigma from family members have the difficulty in recognizing their sexual orientation and conceal it; they may use smartphones to explore the world outside family, connect with LGB communities, and take a break from family discord. However, overuse of smartphones can lead to PSU.

To the best of our knowledge, this study is the first to explore the predictive effects of prepandemic individual and environmental factors on PSU among LGB individuals during the COVID-19 pandemic. Although the severity of PSU was lower at the 4-year follow-up than at baseline, PSU remains a key health concern among LGB individuals and thus warrants attention. The factors significantly associated with PSU should be considered when designing intervention strategies for these individuals. Furthermore, health-care providers should design programs aimed at enabling LGB individuals to develop a mature self-identity and reduce depression. Interventions for the reduction of family and public prejudice toward LGB individuals are also needed.

### Limitations

Our study has some limitations. First, because we collected data from a single source, our findings may be subject to shared-method variance. Moreover, because of the self-report measures used in the present study, our findings could be biased by social desirability. Second, our participants were interested in the follow-up survey; thus, our results may not be generalizable to all LGB individuals. Third, several factors were not evaluated at baseline, such as the types of smartphone activities (e.g., Internet gaming, social media, and dating) and impulsivity; therefore, the predictive effects of these factors on PSU remain undetermined.

## Conclusions

In this study, higher levels of prepandemic PSU, disturbed identity, depressive symptoms, and perceived sexual stigma from family members were associated with a higher level of PSU at follow-up among LGB individuals during the COVID-19 pandemic. The prevention and cure of PSU are crucial. Health-care providers should design programs to help LGB individuals develop a mature self-identity. Further efforts to modify the family and public attitude and prejudice toward transgender LGB individuals are warranted. Furthermore, mental health problems, such as depressive symptoms, among LGB individuals should be explored as the predictors of PSU.

## Data Availability

The data will be available upon reasonable request to the corresponding authors.
